# Molecular targets for diagnostic and intraoperative imaging of pancreatic ductal adenocarcinoma after neoadjuvant FOLFIRINOX treatment

**DOI:** 10.1038/s41598-020-73242-6

**Published:** 2020-10-01

**Authors:** F. A. Vuijk, L. D. A. N. de Muynck, L. C. Franken, O. R. Busch, J. W. Wilmink, M. G. Besselink, B. A. Bonsing, S. S. Bhairosingh, P. J. K. Kuppen, J. S. D. Mieog, C. F. M. Sier, A. L. Vahrmeijer, J. Verheij, A. Fariňa-Sarasqueta, R. J. Swijnenburg

**Affiliations:** 1 Department of Surgery, Leiden University Medical Center, Leiden, The Netherlands; 2 Department of Surgery, Cancer Center Amsterdam, Amsterdam UMC, University of Amsterdam, Amsterdam, The Netherlands; 3 Department of Medical Oncology, Cancer Center Amsterdam, Amsterdam UMC, University of Amsterdam, Amsterdam, The Netherlands; 4 Department of Pathology, Cancer Center Amsterdam, Amsterdam UMC, University of Amsterdam, Amsterdam, The Netherlands

**Keywords:** Cancer imaging, Tumour biomarkers, Pancreatic cancer

## Abstract

Neoadjuvant systemic treatment is increasingly being integrated in the standard treatment of pancreatic ductal adenocarcinoma (PDAC) patients to improve oncological outcomes. Current available imaging techniques remain unreliable in assessing response to therapies, as they cannot distinguish between (vital) tumor tissue and therapy induced fibrosis (TIF). Consequently, resections with tumor positive margins and subsequent early post-operative recurrences occur and patients eligible for potential radical resection could be missed. To optimize patient selection and monitor results of neoadjuvant treatment, PDAC-specific diagnostic and intraoperative molecular imaging methods are required. This study aims to evaluate molecular imaging targets for PDAC after neoadjuvant FOLFIRINOX treatment. Expression of integrin α_v_β_6_, carcinoembryonic antigen cell adhesion molecule 5 (CEACAM5), mesothelin, prostate-specific membrane antigen (PSMA), urokinase-type plasminogen activator receptor, fibroblast activating receptor, integrin α5 subunit and epidermal growth factor receptor was evaluated using immunohistochemistry. Immunoreactivity was determined using the semiquantitative H-score. Resection specimens from patients after neoadjuvant FOLFIRINOX treatment containing PDAC (n = 32), tumor associated pancreatitis (TAP) and TIF (n = 15), normal pancreas parenchyma (NPP) (n = 32) and tumor positive (n = 24) and negative (n = 56) lymph nodes were included. Integrin α_v_β_6_, CEACAM5, mesothelin and PSMA stainings showed significantly higher expression in PDAC compared to TAP and NPP. No expression of α_v_β_6_, CEACAM5 and mesothelin was observed in TIF. Integrin α_v_β_6_ and CEACAM5 allow for accurate metastatic lymph node detection. Targeting integrin α_v_β_6_, CEA, mesothelin and PSMA has the potential to distinguish vital PDAC from fibrotic tissue after neoadjuvant FOLFIRINOX treatment. Integrin α_v_β_6_ and CEACAM5 detect primary tumors and tumor positive lymph nodes.

## Introduction

Pancreatic ductal adenocarcinoma (PDAC) is a devastating malignancy with a five year survival rate of merely 7–9%^1^. This rate reflects the disease’s asymptomatic progression, resulting in advanced-stage disease at the time of diagnosis for the vast majority of patients. Surgical resection combined with systemic treatment offers the only chance for cure. Unfortunately, only 15–25% of patients qualifies for resection^2,3^. Despite careful patient selection and stratification by means of computed tomography (CT), magnetic resonance imaging, and endoscopic retrograde cholangiopancreatography, resection with positive tumor margins (R1) occurs in a substantial proportion of patients (up to 75%)^2,4,5^. Moreover, early recurrences (within six months) after pancreatic resection are reported in 28% of patients, likely due to microscopic tumor deposits at the time of surgery^5^. The clinical relevance of a microscopically radical (R0) resection is further underlined by the two-fold increase in survival time after R0 compared to R1 resection^5–8^.

To improve patient survival and facilitate improved R0 resection rates, neoadjuvant chemotherapy is being implemented increasingly with results being evaluated in several clinical trials, including the Dutch PREOPANC-1 (NTR3709) and PREOPANC-2 trials (NTR7292). However, current imaging modalities struggle to distinguish between vital tumor cells and tumor associated pancreatitis (TAP), therapy induced fibrosis (TIF) and necrosis. As a consequence, 7–13% of pancreatic resections are currently performed for benign conditions^9^, and a large number of patients (up to 92%) is deemed unresectable after neoadjuvant FOLFIRINOX treatment based on conventional imaging have a R0 resection^10^.

With increasing use of potent neoadjuvant therapy, it is of great importance to accurately monitor tumor response to therapy and evaluate surgical resectability after neoadjuvant therapy in order to avoid futile surgical procedures. Both near-infrared fluorescence (NIRF) and positron emission tomography – computed tomography (PET-CT) imaging show promise in providing molecularly targeted imaging solutions to this problem. NIRF imaging is a relatively novel technique that can be used during surgery to discriminate malignant from benign tissue in real time^11^, whereas tumor-specific PET-CT may contribute to improved surgical planning, stratification and diagnosis as well as therapy response monitoring after neoadjuvant treatment. Both modalities exploit tumor-specific tracers (either labeled with a fluorescent protein or radioisotope), targeting biomarkers abundantly present on tumor tissue and absent on (or minimally expressed by) benign or inflamed tissue.

Previous research has shown that [^18^F]FDG-PET/CT is able to influence clinical decision making, but unfortunately with a low specificity of 76% for the detection of PDAC^12^. To enable more specific tumor targeting, our previous immunohistochemical (IHC) studies found both integrin α_v_β_6_ and carcinoembryonic antigen cell adhesion molecule 5 (CEACAM5) to be suitable targets to identify PDAC, distinguishing tumor tissue from TAP or normal pancreatic parenchyma, and also allowing sensitive and specific metastatic lymph node detection^13,14^. Interestingly, after neoadjuvant chemotherapy, α_v_β_6_ expression remained unchanged in vital tumor cells, whereas CEACAM5 expression was reduced^14^. From previous research, we know that not only tumor cells are of influence in cancer progression, the formation of metastases, and the varying response seen after neoadjuvant treatment. Cells of the tumor microenvironment (e.g. cancer-associated fibroblasts and immune cells) are of importance too, and should be considered for both imaging and therapeutic purposes^15,16^. In addition to CEACAM5 and integrin α_v_β_6_, the overexpression of mesothelin^17–23^, prostate-specific membrane antigen (PSMA)^24–28^, urokinase-type plasminogen activator receptor (uPAR)^13,29–31^, fibroblast activation protein alpha (FAP)^32–34^, integrin subunit α5 (ITGA5)^35^ and epidermal growth factor receptor (EGFR) has been described in PDAC tissue, suggesting their candidacy as imaging targets for PDAC.

This study aims to evaluate the immunohistochemical expression of potential molecular imaging targets integrin α_v_β_6,_ CEACAM5, mesothelin, PSMA, uPAR, FAP, ITGA5 and EGFR for the identification of vital residual PDAC and metastatic lymph nodes after neoadjuvant FOLFIRINOX treatment^13,14,17–37^. A graphical overview is shown in Supplementary Fig. 1.

## Results

### Patient characteristics

FFPE tissue from resection specimens of 32 patients treated with neoadjuvant FOLFIRINOX was included. Tissue containing primary tumor and normal pancreatic parenchyma from 32 patients, tumor associated TAP from 16 of these patients, and 24 tumor-positive and 56 tumor-negative lymph nodes were included. Primary tumor and normal pancreatic parenchyma tissue were stained for all eight biomarkers. Tissue containing TAP and lymph nodes were stained only for the four best performing biomarkers (α_v_β_6_, CEACAM5, mesothelin and PSMA), as described in the section below. Patient characteristics are summarized in Table 1.

**Table 1:** Patient characteristics.

		N = 32
Age	Mean (SD)	64.3 (8.8)
Sex	Male	17 (53%)
	Female	15 (47%)
Cycles of neoadjuvant FOLFIRINOX	Median (IQR)	4.5 (2)
ypT	1	4 (13%)
	2	8 (25%)
	3	17 (53%)
	4	3 (9%)
ypN	0	12 (37%)
	1	20 (63%)
ypM	0	31 (97%)
	1	1 (3%)
Differentiation	Good	5 (16%)
	Moderate	16 (50%)
	Poor	8 (25%)
	Missing	3 (16%)
Tumor diameter (mm)	Median (IQR)	30 (23.3)
Total lymph nodes	Median (IQR)	16 (8.8)
Tumor positive lymph nodes	Median (IQR)	1.5 (3)

SD, standard deviation; IRQ, interquartile range; ypT, pathological tumor stage after neoadjuvant therapy; ypN, pathological nodal stage after neoadjuvant therapy; ypM, pathological metastatic stage after neoadjuvant therapy.

### Biomarker expression in primary tumor tissue

**Figure 1:**
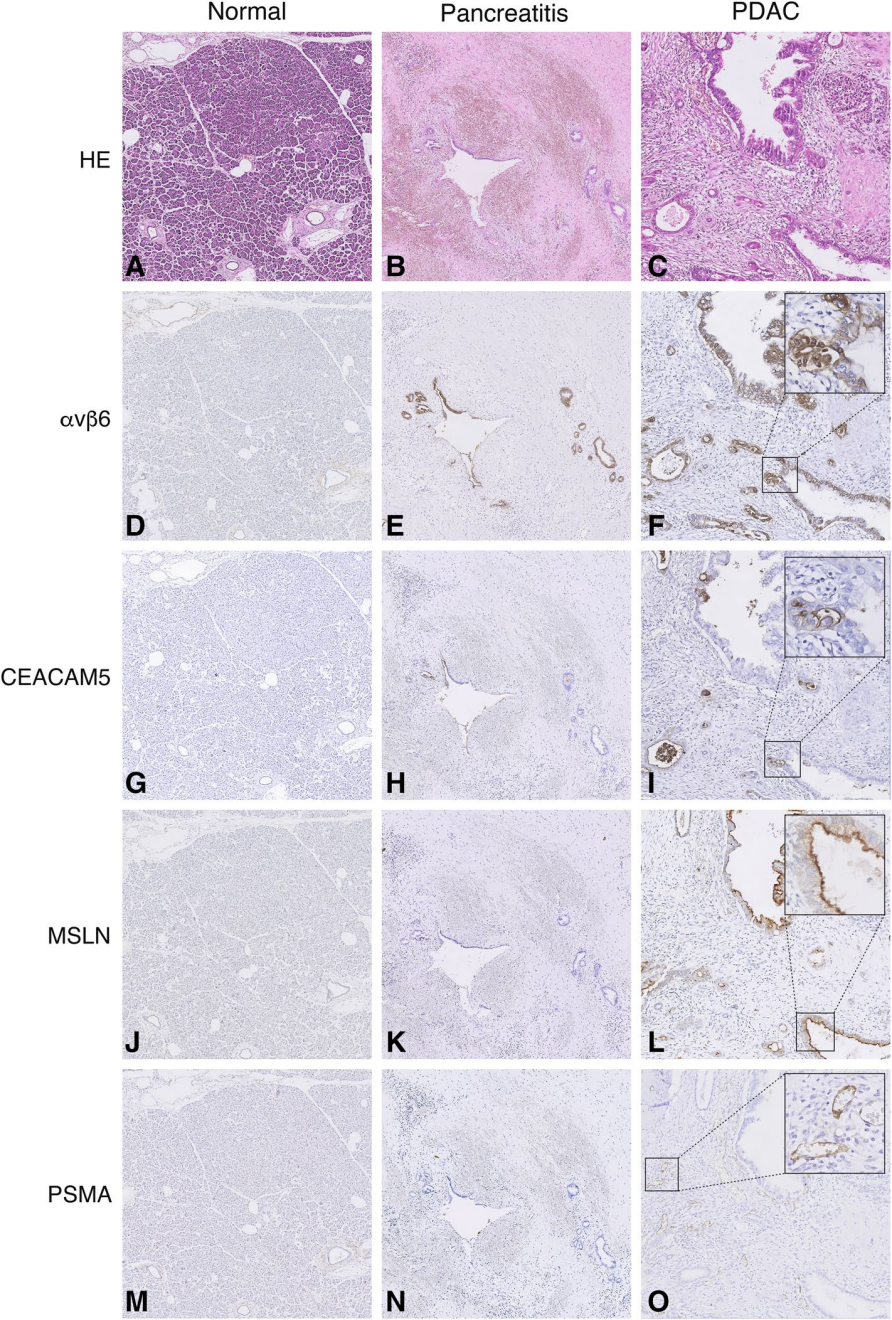
Overview of immunohistochemical staining. Representative images of HE (**A**–**C**), integrin α_v_β_6_ (**D**–**F**), CEACAM5 (**G**–**I**), mesothelin (**J**–**L**) and PSMA (**M**–**O**) expression on normal pancreatic parenchyma, tumor induced pancreatitis and PDAC. All images are at 5 × magnification, zoom images in (**C**,**F**,**I**,**L**,**O**) at 40 × magnification. HE, hematoxylin eosin; α_v_β_6_, integrin α_v_β_6_; CEACAM5, carcinoembryonic antigen cell adhesion molecule 5; MSLN, mesothelin; PSMA, prostate-specific membrane antigen.

All biomarkers, except for ITGA5 and FAP (both mean H-score of 0), were expressed by either tumor- or stromal cells with a median and interquartile range (IQR) tumor H-score of 270 (IQR 50) for αvβ6, 135 (IQR 168) for CEACAM5, 240 (IQR 67) for mesothelin, 60 (IQR 115) for PSMA, and 30 (IQR 50) for uPAR. Integrin α_v_β_6_, CEACAM5, and mesothelin demonstrated membrane-bound tumor cell expression. PSMA was expressed on the endothelium of tumor-associated neovasculature. EGFR showed equal expression in both tumor and normal pancreatic parenchyma. uPAR was expressed very weakly on a low percentage of stromal cells (fibroblasts), but showed high expression by pancreatic islets of Langerhans. Based on these results, uPAR, FAP, ITGA5, and EGFR were excluded from further analyses. Expression patterns of integrin α_v_β_6_, CEACAM5, mesothelin and PSMA are depicted in Fig. 1. Expression patterns of the excluded biomarkers uPAR, FAP, ITGA5 and EGFR are depicted in Supplementary Fig. 2. Results from the immunohistochemical stainings are summarized in Table 2.

**Table 2:** Overview of investigated molecular targets.

Target	Previous research	TNR	Sensitivity lymph node metastases	Specificity lymph node metastases	Other structures expressing target
α_v_β_6_	^13,14,46,51^	4.1	100	100	Duodenum, normal pancreatic parenchyma
CEACAM5	^13,14,36,52,53^	28.5	83	100	
Mesothelin	^17–23^	25.5	67	100	Mesothelium
PSMA	^24–28^	99.4	65	32	Duodenum, germ centers in lymph nodes
EGFR	^13,37,54^	N/A	N/A	N/A	Duodenum, normal pancreatic parenchyma
uPAR	^13,29–31^	N/A	N/A	N/A	Pancreatic islets, neuroendocrine cells, duodenum
FAP	^32–34^	N/A	N/A	N/A	Nerve, muscle, lymphocytes
ITGA5	^35^	N/A	N/A	N/A	Endothelium, duodenum, islet-progenitor acinar cells

TNR, Tumor to Normal ratio (as described in methods); α_v_β_6,_ integrin α_v_β_6_; CEACAM5, carcinoembryonic antigen cell adhesion molecule 5; PSMA, prostate-specific membrane antigen; EGFR, epidermal growth factor receptor; uPAR, urokinase-type plasminogen activator receptor; FAP, fibroblast activating protein; ITGA5, integrin α.

### Tumor-to-normal ratio (TNR)

**Figure 2:**
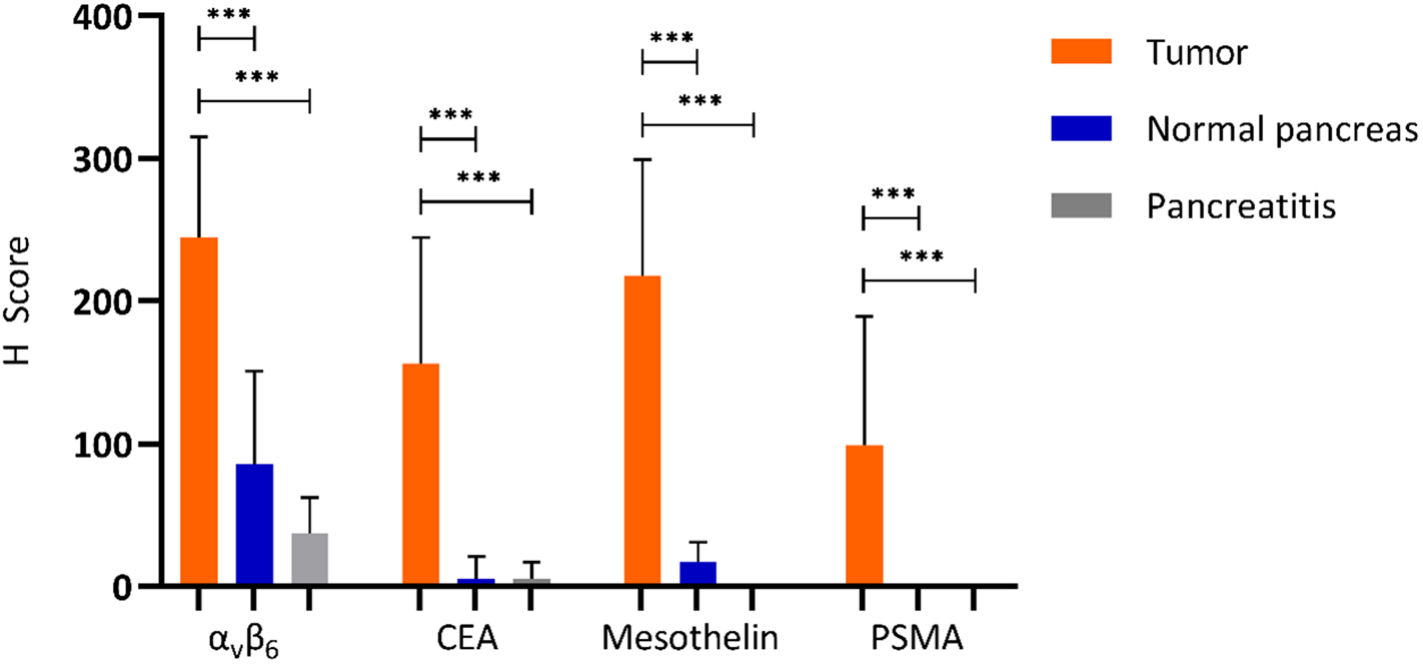
H-scores of selected molecular targets. Representative diagrams of H-scores of integrin α_v_β_6_, CEACAM5, mesothelin and PSMA on tumor (PDAC), normal and tumor induced pancreatitis. H-scores were determined as described in Material and Methods. CEACAM5, carcinoembryonic antigen cell adhesion molecule 5; PSMA, prostate-specific membrane antigen.

Integrin α_v_β_6_, CEACAM5, mesothelin, and PSMA all exhibited significantly higher H-scores on PDAC tissue compared to normal pancreatic parenchyma and TAP (P < 0.001), as depicted in Figs. 1 and 2. Further analysis of H-scores resulted in a TNR of 4.1 for integrin α_v_β_6_, 28.5 for CEACAM5, 25.5 for mesothelin and 99.4 for PSMA.

### Biomarker expression in (therapy induced) fibrosis

Integrin αvβ6, CEACAM5 and mesothelin showed no expression on (therapy induced) fibrotic tissue. PSMA was expressed by neoangiogenic endothelium in close proximity to cancer cells, however not by the cancer cells themselves. After neoadjuvant therapy, capillaries are still present and express PSMA. It is, however, impossible to determine whether these are neoangiogenic capillaries in a former tumor bed, or ‘normal’ capillaries that were never associated with cancer growth.

### Lymph node detection potential

**Figure 3:**
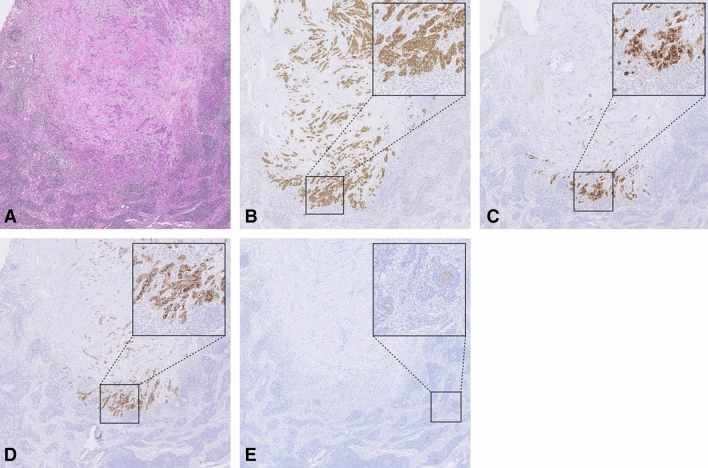
Overview of immunohistochemical stainings on a tumor positive lymph node. Representative images of a metastatic lymph node stained for HE (**A**), integrin α_v_β_6_ (**B**), CEACAM5 (**C**), mesothelin (**D**) and PSMA (**E**). All images are at 2 × magnification, zoom images at 10 × magnification. CEACAM5, carcinoembryonic antigen cell adhesion molecule 5; PSMA, prostate-specific membrane antigen.

Examples of IHC stainings of tumor positive lymph nodes are depicted in Fig. 3. IHC staining identified 24 true positive (TP) and 56 true negative (TN) lymph nodes when staining for integrin α_v_β_6_, 20 TP and 60 TN lymph nodes for CEACAM5, 16 TP and 63 TN lymph nodes for mesothelin and 15 TP and 24 TN lymph nodes for PSMA. This resulted in a sensitivity and specificity of 100% and 100% for integrin α_v_β_6_, 83% and 100% for CEACAM5, 67% and 100% for mesothelin and 65% and 32% for PSMA, respectively, as summarized in Table 3. PSMA staining was only expressed by lymph nodes germinal centers, not by metastatic tumor ducts. An overview of IHC analysis results is provided in Table 3.

**Table 3:** Overview of lymph node detection potential of α_v_β_6_, CEACAM5, mesothelin, and PSMA.

	Sensitivity	Specificity	PPV	NPV
α_v_β_6_	100	100	100	100
CEACAM5	83	100	100	94
Mesothelin	67	100	100	89
PSMA	65	32	33	75

PPV, positive predictive value; negative predictive value; CEACAM5, carcinoembryonic antigen cell adhesion molecule 5; PSMA, prostate-specific membrane antigen.

## Discussion

Our results show significantly higher expression of integrin α_v_β_6_, CEACAM5, mesothelin, and PSMA in PDAC tissue after neoadjuvant therapy as compared to both TAP and normal pancreatic parenchyma. No expression of integrin α_v_β_6_, CEACAM5 and mesothelin was observed in fibrotic tissue, indicating these are potentially suitable targets for vital cancer cell identification after neoadjuvant therapy. In contrast to integrin α_v_β_6_ and CEACAM5, which are also highly sensitive and specific in detecting metastatic lymph nodes, mesothelin and PSMA seem less suitable for this second application.

In line with our previous results, a significant difference in expression of integrin α_v_β_6_ was seen between PDAC tissue and both TAP and normal pancreatic parenchyma. However, in comparison to the other evaluated markers, a low Tumor to Normal Ratio was found due to moderate expression of α_v_β_6_ on normal pancreatic ducts^14^. Moreover, we have previously described integrin α_v_β_6_ expression after neoadjuvant therapy in PDAC as being twice as high in comparison to normal pancreatic parenchyma and four times higher in PDAC compared to TAP^14^. Results from the present study are similar, demonstrating integrin α_v_β_6_ expression in PDAC to be almost three times higher compared to normal pancreatic parenchyma and 7.5 times higher compared to TAP. Before neoadjuvant treatment, CEACAM5 expression was absent in both normal and inflamed pancreatic parenchyma. Interestingly, our previous study described absence of CEACAM5 expression in 2/6 PDAC samples after neoadjuvant treatment^14^. Lack of CEACAM5 expression was seen in only 1/6 patient in this study. Two possible reasons for the reduced expression observed by Tummers et al*.* are tumor heterogeneity, in which CEACAM5 expression is selectively diminished by therapy in a subset of tumor cells, or a selective effect of therapy on the cell genome resulting in clonal evolution^14,38–40^.

Although absolute PSMA expression was lower compared to other molecular targets, specificity for staining tumor associated vessels as well as the contrast seen between normal pancreatic parenchyma and TAP was high (TNR = 99.4). Considering the high sensitivity of both PET and fluorescence imaging (PET 10^–11^ to 10^–12^ M, NIRF 10^–9^ to 10^–12^ M)^41^, the lower absolute expression might not pose a problem. However, considering the nature of targeting, i.e. neoangiogenic endothelial cells, the lack of expression in metastatic lymph nodes would be a limiting factor for PSMA-based targeting. A possible explanation for the absence of PSMA expression in lymph node metastases might lie in the biology of this receptor or lower density of neoangiogenic vessels. PSMA is a type II transmembrane protein upregulated in the neoangiogenesis pathway of solid tumors. Previous clinical and preclinical evidence suggest this pathway is highly activated in primary tumors, however metastatic lymph node development might rely on other pathways. This is demonstrated by the failure of antiangiogenic therapies to completely diminish (lymph node) metastases^42,43^. Previous research shows sprouting angiogenesis is mostly involved in primary tumor angiogenesis, whereas mechanisms such as vessel co-option and intussusception have been implicated in the growth of various cancer metastases, and are possibly also more relevant in lymph node metastases development^44,45^.

As reported by most studies investigating epithelial targets, the exact influence of patchy growth patterns on tracer accumulation and imaging results is uncertain. Although first results from tumor-specific pancreatic carcinoma PET-CT research look promising^46^, future clinical trials will have to provide more insight as to whether heterogenic tracer distribution throughout a larger tumor volume will provide sufficient imaging contrast.

The high expression of integrin α_v_β_6_, CAECAM5, mesothelin, and PSMA, might suggest a functional role of these proteins in the development of PDAC, through for example the β-catenin/wnt signaling pathway, as recently described by Argentiero et al*.*^47^. In line with that, it could be speculated that by suppression of chemokine production by signalling of the previously mentioned proteins, T-cell infiltration can be halted and tumor progression is supported.

Depending on the purpose of imaging, optimal target selection can vary. Integrin α_v_β_6_ and CEA might provide the most versatile imaging targets, offering both primary tumor detection as well as sensitive and specific lymph node imaging. Mesothelin and PSMA, however, are equally suitable for primary detection but lack accuracy in detecting metastatic lymph nodes. Results from previous work from our group demonstrate the feasibility of CEA-targeted imaging in pancreatic cancer patients. Results show tumor specific tracer accumulation and identified previously unseen tumor nodules^36^. The present study shows that FAP, ITGA5 and EGFR are unsuitable targets for molecular imaging of PDAC as FAP and ITGA5 expression was minimal and EGFR was equally expressed by PDAC and normal pancreatic parenchyma. However, a recent study using a FAP targeted PET radioligand, [^68^Ga]-FAPI, was able to detect 51/51 PDAC lesions (mean SUVmax of ~ 10)^32^, EGFR targeting cetuximab-IRDye800 was able to detect 7/7 pancreatic lesions using NIRF imaging^37^, and a recent IHC study described strong ITGA5 expression in the tumor stroma of 66% out of 137 primary PDAC samples (without neoadjuvant treatment)^35^. These results put the limited translational value of IHC studies in predicting clinical imaging results into perspective, and demonstrate that more than just receptor expression is involved in reaching successful tracer uptake in tumor tissue. Future animal studies will have to provide more information on the success of targeting these biomarkers for imaging.

Possible limitations of this study include a relatively small sample size, semi-quantitative analysis of IHC results and the lack of knowledge regarding biomarker expression in these patients before neoadjuvant therapy. Direct comparison before and after therapy was unfortunately not possible, as no pre-operative tissue was available. Nonetheless, due to previous work within our group and the fact that only targets with known overexpression were investigated in a substantial number of patients, we feel confident that expression levels in these tumors represent the general population and provide clinically relevant information.

In conclusion, integrin α_v_β_6_, CEACAM5, mesothelin, and PSMA are potential suitable targets for both pre-operative as well as intraoperative molecular imaging before and after neoadjuvant FOLFIRINOX treatment, as will have to be confirmed by future clinical imaging studies. Using PET-CT, NIRF, or other molecular imaging modalities, both integrin α_v_β_6_ and CEACAM5 show most promise as molecular targets for the imaging of PDAC and metastatic lymph nodes, as is currently being further investigated in the PANSCAN trial and other clinical studies^48^.

## Methods

### Patient and material selection

Patients admitted to the Amsterdam UMC (location AMC) diagnosed with PDAC and treated with neoadjuvant FOLFIRINOX treatment (consisting of folinic acid, 5′-fluorouracil, irinotecan, and oxaliplatin) were retrospectively included. After surgical resection, representative formalin-fixed paraffin-embedded (FFPE) tissue blocks containing tumor, normal pancreatic parenchyma, and TAP, as well as tumor positive and negative lymph nodes, were selected and obtained from the Department of Pathology (Amsterdam UMC, location AMC). Clinicopathologic characteristics were obtained from medical records. The need for ethical approval and individual consent was waived by the Institutional Medical Ethics Committee of the Amsterdam UMC, and this study conducted in accordance with the Declaration of Helsinki.

### Immunohistochemistry

FFPE tissue sections at four µm thickness were sliced and stained for integrin α_v_β_6_, CEACAM5, mesothelin, PSMA, uPAR, FAP, ITGA5 and EGFR. After deparaffinization in xylene and rehydration in a stepwise series of alcohol solutions, endogenous peroxidase activity was blocked with 0.3% hydrogen peroxide in water for 20 min. Antigen retrieval was performed as described in Supplementary Table 1. Following antigen retrieval, slides stained for FAP were incubated for 10 min with Protein Block (Dako, Glostrup, Denmark). All slides were incubated overnight at room temperature with primary antibodies (Supplementary Table 1). Slides were washed in phosphate-buffered saline (PBS) and incubated for 30 min at room temperature with an HRP-labelled secondary antibody (anti-mouse, anti-rabbit (Envision, Dako, Glostrup, Denmark) or anti-donkey (Invitrogen, Carlsbad, USA)). After being rinsed with PBS, immunoreactions were visualized using DAB substrate buffer (Dako, Glostrup, Denmark) for ten minutes and counterstained using Mayer’s hematoxylin for 30 s. After dehydration at 37 °C, the slides were mounted with PERTEX® (Leica Microsystems, Wetzlar, Germany).

### Evaluation of immunoreactivity

Evaluation of immunoreactivity was performed by two independent pathologists in tandem (A.F.S. and J.V.) and was conducted using the semi-quantitative H-score^49,50^. Consensus was reached for all patients. This score takes into account both staining intensity and percentage of cells stained and is used by multiplying the staining intensity (0, 1, 2, or 3) by the percentage of cells expressing the target at this intensity (0–100%), resulting in a score ranging from 0 to 300. As a result, higher H-scores indicate more intense staining in a higher percentage of cells.

To define the contrast that a molecular target provides in distinguishing PDAC from normal pancreatic parenchyma or TAP, the Tumor to Normal Ratio (TNR) was established. The TNR was calculated by dividing the Tumor H-score by the Normal H-score (average H-score of normal pancreatic parenchyma and TAP. The H-score for Normal was defined as 1 when no expression was seen in TAP or normal pancreatic parenchyma.

The lymph node detection potential was evaluated by calculating sensitivity, specificity, positive predictive value (PPV), and negative predictive value (NPV) of selected biomarkers to correctly identify tumor positive lymph nodes. Sensitivity was calculated by dividing the true positive lymph nodes (TPLN) by the sum of TPLN and the false-negative lymph nodes (FNLN). Specificity was calculated by dividing the true negative lymph nodes (TNLN) by the sum of the TNLN and false-positive lymph nodes (FPLN). PPV was calculated by dividing the TPLN by the sum of TPLN and FPLN. NPV was calculated by dividing the TNLN by the sum of the TNLN and FNLN.

### Statistical analysis

Statistical analysis was performed using SPSS version 25 (IBM SPSS, Inc., Chicago, USA) and GraphPad Prism 8 (GraphPad Software, Inc., San Diego, USA). Continuous descriptive data respecting a Gaussian distribution were displayed as mean (standard deviation), or median (interquartile range) when non-parametric. Categorical data were displayed as frequencies and percentages. H-scores were compared using the Kruskal Wallis one way ANOVA test with post hoc Bonferroni correction for multiple testing. Results were considered significant when *p* < 0.05.

## Supplementary information

Supplementary Figures.

Supplementary Table 1.

## Data Availability

The datasets generated and/or analyzed during the current study are available from the corresponding author on reasonable request.
