# Supplementary material for: Molecular targets for diagnostic and intraoperative imaging of pancreatic ductal adenocarcinoma after neoadjuvant FOLFIRINOX treatment
Source: Sci Rep. 2020 Oct 1;10:16211. doi: 10.1038/s41598-020-73242-6 (PMC7529886; doi:10.1038/s41598-020-73242-6)
Supplement: Supplementary file 1 — Supplementary Figures. [file 41598_2020_73242_MOESM1_ESM.pdf]

Supplementary Material

Molecular targets for diagnostic and intraoperative imaging of pancreatic ductal adenocarcinoma after neoadjuvant FOLFIRINOX treatment.

F.A. Vuijk[1], BSc., L.D.A.N. de Muynck[1], MSc., L.C. Franken[2], M.D., O.R. Busch[2], M.D., Ph.D., J.W. Wilmink[3], M.D., Ph.D., M.G. Besselink[2], M.D., Ph.D., B.A Bonsing[1], M.D., Ph.D., S.S. Bhairosingh[1], BSc., P.J.K. Kuppen[1], Ph.D., J.S.D. Mieog[1], M.D., Ph.D., C.F.M Sier[1], Ph.D., A.L. Vahrmeijer[1], M.D., Ph.D., J. Verheij[4], M.D., Ph.D., A. Fariňa-Sarasqueta[4], M.D., Ph.D., R.J. Swijnenburg[2*], M.D., Ph.D.

Affiliations

[1] Department of Surgery, Leiden University Medical Center, the Netherlands.

[2] Department of Surgery, Cancer Center Amsterdam, Amsterdam UMC, University of Amsterdam, the Netherlands.

[3] Department of Medical Oncology, Cancer Center Amsterdam, Amsterdam UMC, University of Amsterdam, the Netherlands.

[4] Department of Pathology, Cancer Center Amsterdam, Amsterdam UMC, University of Amsterdam, the Netherlands.

Corresponding author: Dr. R.J. Swijnenburg, M.D., Ph.D.
Department of Surgery, Cancer Center Amsterdam
Amsterdam UMC, University of Amsterdam
Meibergdreef 9, 1105 AZ Amsterdam
The Netherlands
Mail: r.j.swijnenburg@amsterdamumc.nl

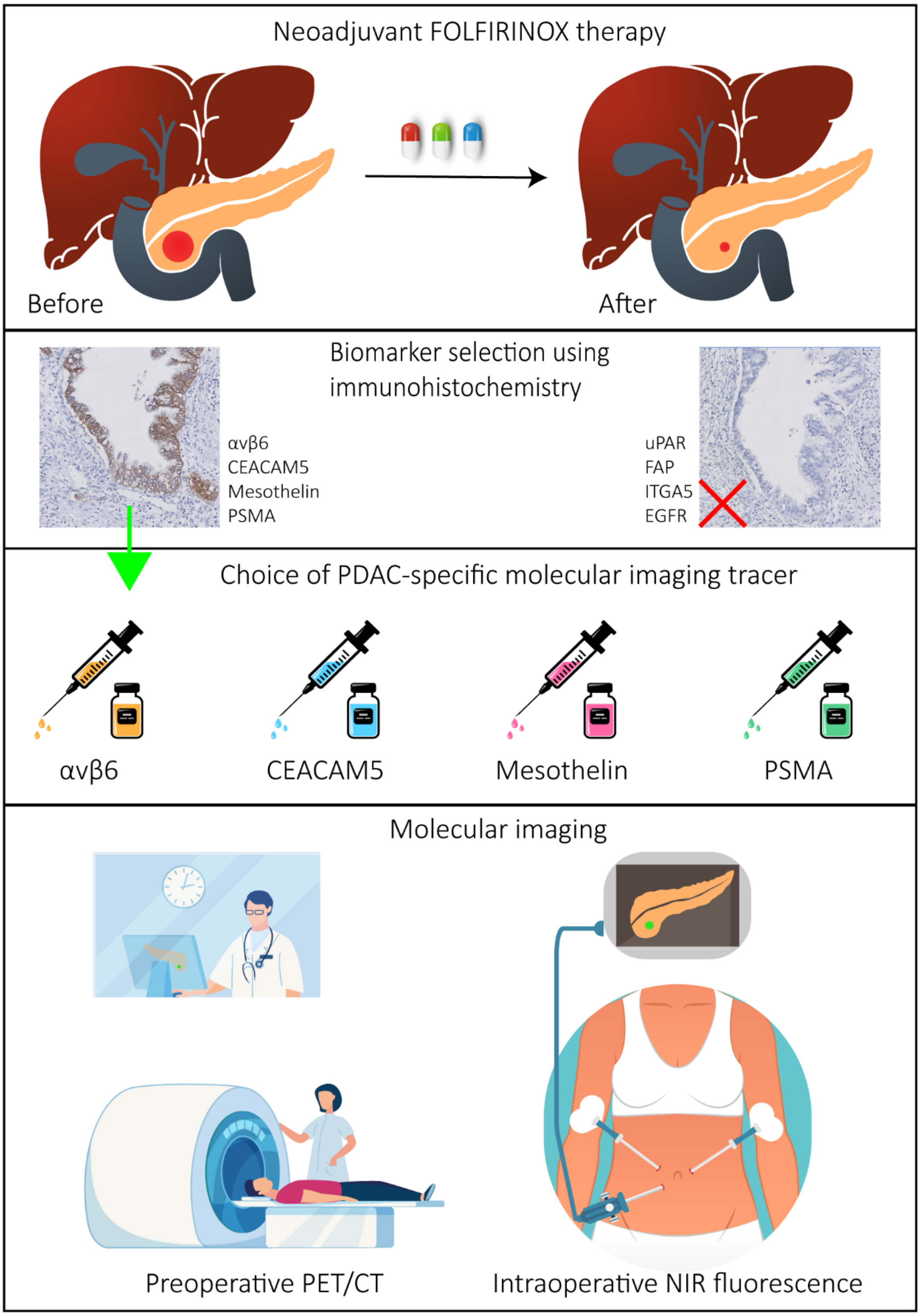

Supplementary Figure 1. Graphical study overview.

Graphical representation of this immunohistochemical study.

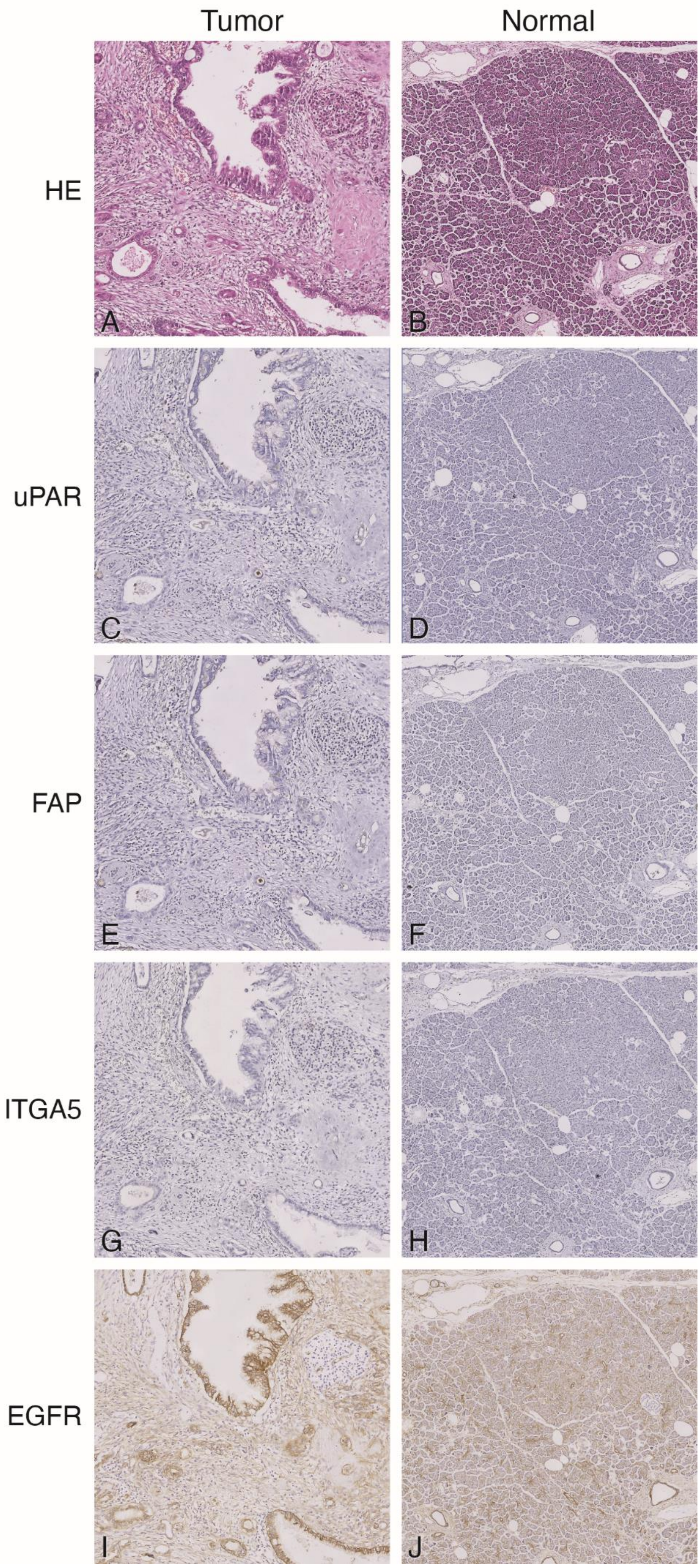

Supplementary Figure 2. Overview of immunohistochemical staining of EGFR, uPAR, FAP and ITGA5.

Representative images of HE, EGFR, uPAR, FAP and ITGFA expression on normal pancreatic parenchyma and PDAC. All images are at 10x magnification. Abbreviations: HE, hematoxylin eosin; EGFR, epidermal growth factor receptor; uPAR, urokinase-type plasminogen activator receptor; FAP, fibroblast activating protein alpha; ITGA5, integrin subtype α5.
