# Supplementary material for: Molecular targets for diagnostic and intraoperative imaging of pancreatic ductal adenocarcinoma after neoadjuvant FOLFIRINOX treatment
Source: Sci Rep. 2020 Oct 1;10:16211. doi: 10.1038/s41598-020-73242-6 (PMC7529886; doi:10.1038/s41598-020-73242-6)
Supplement: Supplementary file 2 — Supplementary Table 1. [file 41598_2020_73242_MOESM2_ESM.docx]

**Supplementary Table 1. Monoclonal Antibody Selection and Immunohistochemical Protocol.**

| **Antibody** | **Clone number** | **Host/isotype** | **Used concentration** | **Antigen retrieval** | **Vendor** |
| --- | --- | --- | --- | --- | --- |
| α_v_β_6_ | 6.2A1 | Mouse | 0.5 µg/ml | 0.125% and 0.4% pepsin at 37⁰C | Biogen Idec MA Inc., Cambridge, USA. |
| CEACAM5 | CI-P83-1 | Mouse | 0.2 µg/ml | Citrate buffer 95⁰C* | Santa Cruz Biotechnology, Inc., Dallas, USA |
| EGFR | E30 | Mouse | 2.86 µg/ml | 0.4% pepsin and 1N HCl at 95⁰C | Dako, Glostrup, Denmark |
| uPAR | ATN-617 | Mouse | 1.2 µg/ml | Citrate buffer 95⁰C* | Kindly provided by prof. Andrew P. Mazar |
| FAP | AF3715 | Donkey | 2 µg/ml | Tris-EDTA buffer (pH 9.0) at 95⁰C | Invitrogen, Carlsbad, USA |
| ITGA5 | HPA002642 | Rabbit | 0.2 µg/ml | Citrate buffer 95⁰C* | Atlas Antibodies, Bromma, Sweden |
| PSMA | 3E6 | Mouse | 1.64 µg/L | Tris-EDTA buffer (pH 9.0) at 95⁰C | Dako, Glostrup, Denmark |
| Mesothelin | MN-1 | Mouse | 0.67 µg/ml | Tris-EDTA buffer (pH 9.0) at 95⁰C | Rockland Immunochemicals, Inc., Limerick, UK |

Abbreviations: CEACAM5, carcinoembryonic antigen cell adhesion molecule 5; EGFR, epidermal growth factor receptor; uPAR, urokinase-type plasminogen activator receptor; FAP, fibroblast activating receptor; ITGA5, integrin α5; PSMA, prostate-specific membrane antigen.
* In PT-Link module (Agilent, Santa Clara, USA).
